# Startle Disease: New Molecular Insights into an Old Neurological Disorder

**DOI:** 10.1177/10738584221104724

**Published:** 2022-06-25

**Authors:** Natascha Schaefer, Robert J. Harvey, Carmen Villmann

**Affiliations:** 1 Institute of Clinical Neurobiology, University Hospital, Julius-Maximilians-University of Würzburg, Würzburg, Germany; 2 School of Health and Behavioural Sciences, University of the Sunshine Coast, Maroochydore DC, Australia; 3 Sunshine Coast Health Institute, Birtinya, Australia

**Keywords:** glycine receptor, glycine transporter, startle disease, protein-protein interactions, maturation, trafficking, functional inhibition

## Abstract

Startle disease (SD) is characterized by enhanced startle responses, generalized muscle stiffness, unexpected falling, and fatal apnea episodes due to disturbed feedback inhibition in the spinal cord and brainstem of affected individuals. Mutations within the glycine receptor (GlyR) subunit and glycine transporter 2 (GlyT2) genes have been identified in individuals with SD. Impaired inhibitory neurotransmission in SD is due to pre- and/or postsynaptic GlyR or presynaptic GlyT2 dysfunctions. Previous research has focused on mutated GlyRs and GlyT2 that impair ion channel/transporter function or trafficking. With insights provided by recently solved cryo–electron microscopy and X-ray structures of GlyRs, a detailed picture of structural transitions important for receptor gating has emerged, allowing a deeper understanding of SD at the molecular level. Moreover, studies on novel SD mutations have demonstrated a higher complexity of SD, with identification of additional clinical signs and symptoms and interaction partners representing key players for fine-tuning synaptic processes. Although our knowledge has steadily improved during the last years, changes in synaptic localization and GlyR or GlyT2 homeostasis under disease conditions are not yet completely understood. Combined proteomics, interactomics, and high-resolution microscopy techniques are required to reveal alterations in receptor dynamics at the synaptic level under disease conditions.

## Introduction

Startle disease (SD; hyperekplexia, stiff baby syndrome, OMIM 149400) is a rare neurological disease known since the 1960s. This rare neuromotor disorder affects spinal inhibitory motor circuits, causing increased muscle tone and exaggerated startle reflexes. However, not every excessive startle reaction is caused by SD. Three main characteristics determine SD: severe muscle stiffness in neonates, enhanced startle reflexes in response to unexpected acoustic or tactile stimuli, and generalized stiffness following the startle response without any possible voluntary movement (Tijssen and others 2002). However, the severity of symptoms can vary considerably among affected individuals, ranging from generalized muscle stiffness to unprotected falls and injury or even seizures and/or fatal apnea episodes (Mine and others 2015).

**Figure 1.**
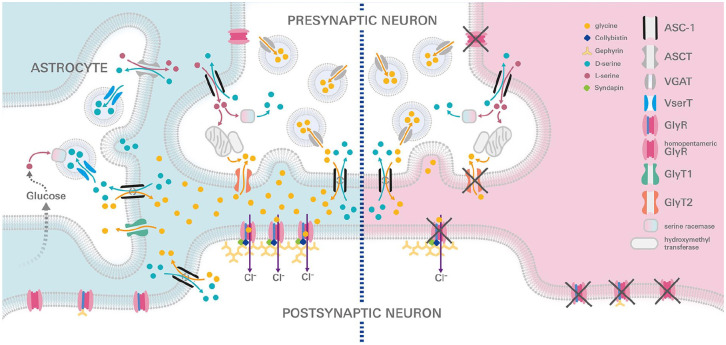
Inhibitory synapse under healthy and disease conditions. The inhibitory glycinergic synapse is shown under two conditions: healthy (left, blue) and startle disease (right, red). Glycine receptors (GlyRs) are localized in a homomeric α subunit configuration at the presynaptic site, whereas heteromeric αβ GlyRs are present at synaptic and extrasynaptic sites bound to the scaffold protein gephyrin at the membrane of postsynaptic neurons. Following glycine release from the presynaptic compartment, it binds postsynaptically to the GlyRs, which upon opening leads to a chloride ion influx into the postsynaptic neuron. Glycine transporter 2 (GlyT2), the presynaptic glycine transporter, enables glycine recycling. Glycine is packed into vesicles and transported by the vesicular GABA transporter (VGAT; also known as VIAAT [vesicular inhibitory amino acid transporter]). The alanine-serine-cysteine transporter 1 (ASC-1) and the neutral amino acid transporter (ASCT) are important for D-serine clearance from the synaptic cleft; however, the ASC-1 also has a high affinity for glycine and L-serine. D-serine is formed from L-serine by serine racemase. VserT is the astrocytic vesicular D-serine transporter.

**Figure 2.**
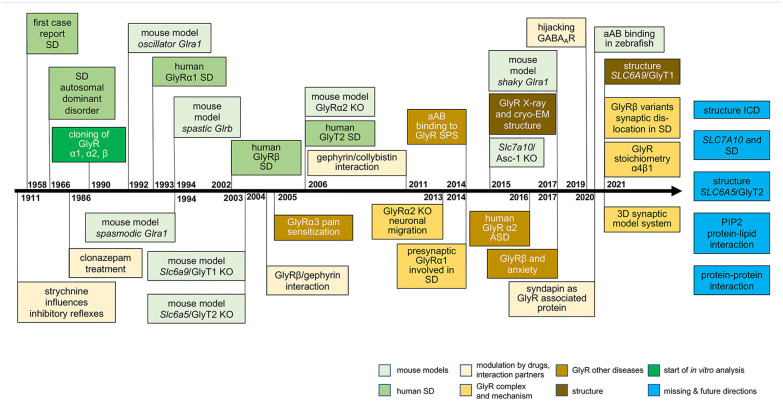
Timeline of discoveries unraveling a molecular understanding of glycinergic transmission in SD. The identification of startle disease (SD) goes back to the 1960s. The timeline describes the discoveries of molecular candidates within the last 30 y involved in the pathology of SD (human SD = green boxes). Following the description of candidate genes, the cloning of glycine receptor subunits (green box) and the characterization of spontaneous mouse models (light green boxes = mouse models) associated with SD helped to understand the causality between receptor mutations and disease phenotype. Knockout (KO) mouse models (light green boxes = mouse models) have further provided impact to novel targets such as glycine transporter 2 (GlyT2) and possibly alanine-serine-cysteine transporter 1 (Asc-1); light brown boxes = glycine receptor [GlyR] other diseases. At the molecular level, in vitro mutagenesis studies exhibited alterations in protein expression level and functionality. With the help of the solved structures of GlyRs by X-ray or cryo–electron microscopy, functional changes could have been better explained by structural alterations upon mutation (brown boxes = structure). The complexity of the disease became apparent with novel single mutations or compound heterozygous cases not showing obvious alterations of GlyR expression or function (GlyR complex and mechanism = yellow boxes). Disease complexity increased by the contribution of protein-protein interactions (modulation by drugs, other proteins = light yellow boxes) inside the neurons, as well as the identification of presynaptic GlyRs and mechanisms such as hijacking of GABA_A_ receptors by GlyR mutants. Possible future directions are shown on the right end (missing and future directions = blue boxes).

The major causes of SD are genetic defects in *GLRA1* and *GLRB*, encoding the postsynaptic glycine receptor (GlyR) α1 and β subunits, or *SLC6A5*, encoding the glycine transporter 2 (GlyT2; Figs. 1, 2; Rees and others 2006). Importantly, symptomatic pharmacotherapy with benzodiazepines (e.g., clonazepam) is effective in patients with mutations in GlyR subunits or GlyT2 (Thomas and others 2010).

Mutations in GlyRs or GlyT2 reduce glycinergic neurotransmission, thus affecting the balance between excitation and inhibition in brainstem and spinal cord circuits. A reduction in the activity of glycinergic interneurons in the spinal cord results in increased muscle contraction and stiffness. Moreover, the massive and uncontrolled startle reaction observed in SD is due to an enhanced startle reflex mediated by brainstem nuclei (Schaefer and others 2012). Glycinergic dysfunction in brainstem nuclei is also thought to be responsible for neonatal apnea episodes or in rare cases of sudden infant death (Bode and Lynch 2014).

**Figure 3.**
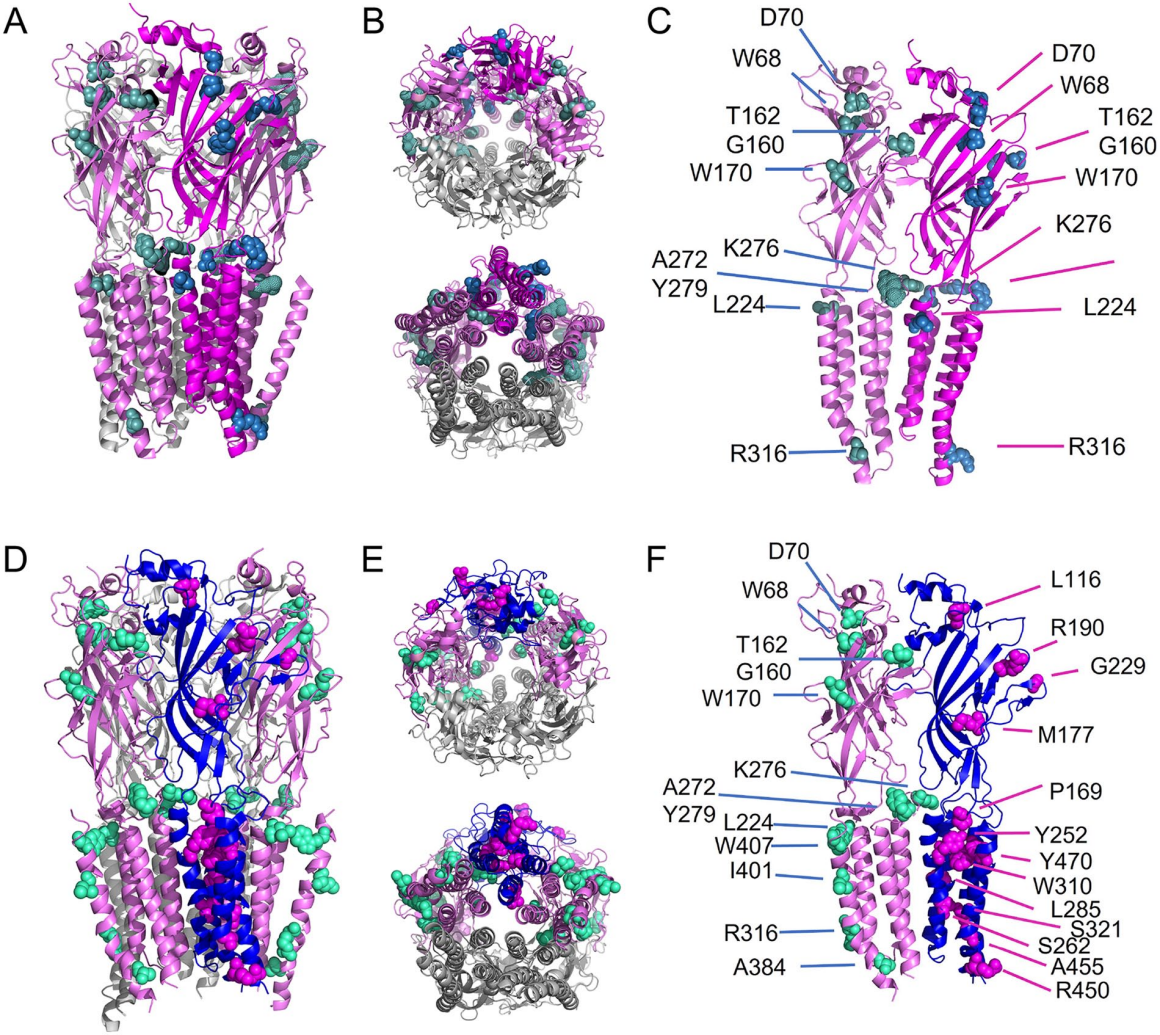
Visualization of novel startle disease (SD) mutations in homomeric and heteromeric glycine receptors (GlyRs). Structure of homo- and heteropentameric GlyRs including mutations associated with startle disease. Structures of homopentameric GlyR based on GlyRα3 (5VDH; Huang and others 2015) and structures of heteropentameric GlyR used the 4α1:1β stochiometry (7MLU; Zhu and Gouaux 2021). (A) GlyRα1 homopentamer with residues affected in SD (green and blue; Table 1): α1+ subunit in light magenta (left front), α1– subunit in dark magenta (right front), and additional subunits in the back (light magenta and gray). (B) Homopentameric GlyR, top and bottom view. (C) Only two adjacent subunits are shown (α1+ and α1– interface) with affected residues marked by amino acid numbers (Table 2). (D) GlyR heteropentamer with one β subunit (blue) and four α1 subunits (two depicted in light magenta and two in gray), GlyRβ mutations (magenta), and GlyRα1 mutations (light green) (Tables 2 and 3). Heteropentameric GlyR: (D) side view, (E) top and bottom view, (F) α1+ subunit (light magenta) and β– subunit (blue) with marked residues affected in SD. Note that in the heteromeric GlyR, the α1^A384T^ mutation is included due the use of the template provided by Zhu and Gouaux (2021).

GlyRs enable fast synaptic inhibition in the central nervous system and belong to the superfamily of cys-loop receptors. Cys-loop receptors form pentameric ion channels that share a common disulfide bridge in the ligand-binding extracellular domain (ECD). The ECD is followed by four transmembrane (TM) domains connected by loop structures (TM1-2, TM2-3, and TM3-4) and a short extracellular C-terminus (Schaefer and others 2018a). The large intracellular domain (ICD) exhibits the highest variability among GlyRs and determines subunit-specific properties, such as phosphorylation, ubiquitination, and interaction with intracellular binding partners (Langlhofer and others 2020). GlyRs are activated by glycine, β-alanine, and taurine and antagonized by strychnine, an alkaloid from *Nux vomica* (Schaefer and others 2012). GlyRs can form homomeric (α1-4) or heteromeric (αβ) receptors. Cryo-EM (electron microscopy) structures suggest a stoichiometry of native GlyRs with 4α:1β (Fig. 3; Yu and others 2021; Zhu and Gouaux 2021).

GlyT2 belongs to the family of neurotransmitter sodium symporters and regulates the concentration of synaptic glycine via reuptake to presynaptic terminals. GlyT2 is a 12-TM protein with intracellular N- and C-termini (Kristensen and others 2011). Alterations in transport mechanisms upon GlyT2 mutations in SD have been studied by homology modeling and molecular dynamic simulations using structures of the bacterial leucine transporter, the dopamine transporter, the human serotonin transporter, and the recently solved X-ray structure of the human GlyT1 transporter (Carta and others 2012; Shahsavar and others 2021).

## Important Players at the Glycinergic Synapse

Dysfunctional glycinergic synapses in the spinal cord and brainstem underlie SD pathology at the molecular level. Excited ventral spinal cord motoneurons fire action potentials to the neuromuscular endplate with signal propagation via cholinergic pathways to the muscle. This process is controlled by feedback inhibition via inhibitory interneurons (Schaefer and others 2012). Our classical view of glycinergic synapses is based on presynaptic packaging of glycine into vesicles mediated by the vesicular inhibitory amino acid transporter (VIAAT/VGAT), GlyT2 as a pivotal transporter for glycine reuptake to the presynaptic compartment, and the postsynaptic chloride-permeable GlyR anchored by the scaffolding protein gephyrin to the cytoskeleton (Fig. 1; Chapdelaine and others 2021). However, this model has been extended by the existence of homomeric presynaptic GlyRs, which contribute to SD pathology; the presynaptic Asc-1 (alanine-serine-cysteine transporter 1), which is important for glycine and serine transport; the presence of extrasynaptic GlyRs, also coupled to gephyrin; and a possibly modulatory role of the interaction partner syndapin I in SD (Figs. 1 and 2, Table 1; Chapdelaine and others 2021; Langlhofer and others 2020; Safory and others 2015; Xiong and others 2014).

**Table 1.** Important Players at Glycinergic Synapses in Humans.

Gene	Protein	Protein Full Name
*GLRA1*	GlyRα1	Glycine receptor α1 subunit
*GLRA2*	GlyRα2	Glycine receptor α2 subunit
*GLRA3*	GlyRα3	Glycine receptor α3 subunit
*GLRB*	GlyRβ	Glycine receptor β subunit
*SLC6A9*	GlyT1	Glycine transporter 1
*SLC6A5*	GlyT2	Glycine transporter 2
*SLC7A10*	ASC-1	Alanine-serine-cysteine transporter 1
*CTNNB1*	β-catenin	β-Catenin
*GPHN*	geph	Gephyrin
*PACSIN1*	Sdp I	Syndapin I
*ARHGEF9*	Cb	Collybistin
*SLC32A1*	VIAAT/VGAT	Vesicular inhibitory amino acid transporter

## Classical View of Pathological Mechanisms in SD

Although known since the 1960s, the genetic basis for SD was defined with the discovery of mutations in *GLRA1* (Shiang and others 1993) encoding the inhibitory GlyRα1 subunit. Since then, >50 mutations in *GLRA1* have been reported, making mutations in this gene the most common cause of SD, followed by *SLC6A5* and *GLRB*. The first mutations in *GLRB* were discovered by Rees and others (2002), while mutations in *SLC6A5* were first reported in 2006 (Rees and others 2006), with further cohort studies reported in 2012 (Carta and others 2012; Gimenez and others 2012; Fig. 2, Table 1). At the molecular level, mutations have been classified into dominant, recessive, and gain of function (Bode and Lynch 2014).

### Dominant Mutations

Most dominant mutations (e.g., P250T, V260M, Q266H, S267N, R271Q) are in fact loss-of-function mutations, as they uncouple ligand binding from channel gating (Bode and Lynch 2014). Gating of the GlyR was more precisely defined with the cryo-EM structure of the homomeric zebrafish GlyRα1. Extensive interactions at subunit interfaces near the ECD–TM domain boundary were identified. Those interactions involve β1-β2, β8-β9, the cys-loop, pre-TM1/TM1, and the TM2-3 loop (Du and others 2015). Moreover, the structural information has been an importfant breakthrough for the interpretation of the physiological defects obtained for various SD mutations. The GlyRα1^P250T^ variant was originally described as a gating variant, arguing that the introduction of a threonine leads to loss of an angular polypeptide structure, thereby destabilizing open channel conformations and explaining the fast GlyR desensitization upon glycine application (Saul and others 1999). The cryo-EM structures of GlyRα1 further revealed the importance of P250 at position −2 of the ion channel pore as it represents a physical site of constriction in the glycine-bound open state, thus allowing the permeation of the hydrated chloride ions (Du and others 2015).

### Recessive Mutations

Although recessive mutations result in loss of function, the major difference is that dominant mutations do not affect surface expression, while many recessive mutants result in altered GlyR trafficking, lower cell membrane expression, decreased ligand binding or splice site, nonsense, frameshift, or deletions that result in protein truncation (Schaefer and others 2015; Fig. 3, Tables 2 and 3). Every generated GlyR needs to pass several maturation and quality control mechanisms to reach the cellular surface. SD mutations can therefore lead to failures in maturation or quality control. Maturation of the GlyR involves posttranslational folding and glycosylation in the endoplasmic reticulum (ER). The glycosylation state of the GlyR represents a prerequisite for pentameric assembly and is necessary for ER exit. Moreover, there are chaperone proteins that escort surface proteins from the ER to the Golgi (e.g., ERGic-53; Schaefer and others 2018a). Disrupted neuronal ER-Golgi trafficking was identified for several recessive GlyRα1 subunit SD mutants (Schaefer and others 2015). Recessive mutations are distributed across the whole GlyRα1 and GlyRβ subunits. The majority of GlyRβ mutations also result in reduced surface trafficking, increased glycine EC_50_ values, and/or decreased maximal responses of α1β GlyRs (Chung and others 2013; James and others 2013; Piro and others 2021; Fig. 3, Table 3).

**Table 2.** Mutations in *GLRA1* Encoding the GlyRα1 Subunit and Functional Alterations (since 2015).

				Defect		
Mutation	Compound Heterozygous	Inheritance	Location in GlyR	Biogenesis	Function	References
I43F		Recessive	ECD β1		Gain of function	Zhang and others 2016
W68C	R316X	Recessive	ECD β2	Trafficking	Nonfunctional	Schaefer and others 2015
D70N	W407N / R316X	Recessive	ECD β2	Trafficking	Nonfunctional	Schaefer and others 2015; Zhang and others 2020
R72H/C		Recessive	ECD β2-β3	Trafficking	Nonfunctional	Schaefer and others 2015
G160R		Dominant	ECD β7-β8		Change in glycine EC_50_	Schaefer and others 2015
T162M		Recessive	ECD β7-β8	Trafficking	Change in glycine EC_50_	Schaefer and others 2015
W170S		Recessive	ECD β8		Impaired zinc inhibition	Zhang and others 2016
L224X	I401N	Recessive				Zhan and others 2020
A272P		Dominant	TM2			Mine and others 2015
K276E/Q		Dominant / K276Q de novo	TM2-3		Reduced glycine sensitivity and open probability	Mine and others 2015
Y279C/S/X		Dominant / Y279X recessive	TM2-3		Reduced glycine sensitivity and whole cell current magnitude	Mine and others 2015
R316X	W68C, D70N, R392H	Recessive	TM3-4	Trafficking	Nonfunctional	Mine and others 2015; Schaefer and others 2015; Zhang and others 2020
A384P	R392H	Recessive	TM3-4		Functional, desensitization impaired	Wang and others 2018
R392H	R252H, A384P, R316X	Recessive	TM4	Trafficking	Functional	Mine and others 2015
I401N	L224X	Recessive	TM4		Weird laughing	Zhan and others 2020

Amino acid residues refer to mature protein.

GlyR = glycine receptor.

**Table 3.** Mutations in *GLRB* Encoding the GlyRβ Subunit and Functional Investigations.

				Defect		
Mutation	Compound Heterozygous	Inheritance	Location in GlyR	Biogenesis	Function	References
F-191fsX3		Recessive	ECD			Chung and others 2013
∆ex1-8		Recessive	ECD			Chung and others 2013
∆ex5 and S176RfsX6		Recessive	ECD			Chung and others 2013
E24X		Recessive	ECD N-terminus			Chung and others 2013
R50X	Q216fsX222	Recessive	ECD α1-β1			Mine and others 2013
L116P		Recessive	ECD β6-β7 (Cys loop)	Reduced surface expression	Functional	Chung and others 2013
P169L				Reduced surface expression		Chung and others 2013
M177R		Recessive	ECD β7		Increased EC_50_, decreased I_max_	James and others 2013
R190X		∆S262	Recessive ECD β8		Reduced chloride ion influx	Chung and others 2013; Gupta and others 2020
Q216fsX222		R50X	Recessive ECD			Mine and others 2013
G229D	∆ex5	Recessive	ECD β10			Rees and others 2002
IVS5+5G>A			Splice site			James and others 2013
Y252S			TM1	Reduction in β positive synapses	Increased EC_50_	Piro and others 2021
∆S262	R190X	Recessive	TM1	Reduced surface expression	Reduced chloride ion influx	Chung and others 2013
L285R		Recessive	TM2	Reduced surface expression	Reduced peak currents	James and others 2013
W310C		Recessive	TM2-3	Reduced surface expression	Reduced I_max_	James and others 2013
S321F	In4 (c.298-1G<A)	Recessive	TM3	β aggregates increased in area and perimeter		Piro and others 2021
R450X		Recessive	TM3-4	Reduced surface expression	Functional	Chung and others 2013
A455P			TM4	Reduction in β positive synapses	Gain of function	Piro and others 2021
Y470C		Dominant	TM4	Reduced surface expression	Functional	Chung and others 2013

Amino acid residues refer to mature protein.

GlyR = glycine receptor.

GlyT2 missense mutations typically affect subcellular GlyT2 localization, glycine uptake, or both, with some mutations affecting predicted glycine, Na^+^, and Cl^-^ binding sites (Carta and others 2012; Gimenez and others 2012; Rees and others 2006). A study on the recurrent Y705C mutation in TM domain 11, found in eight individuals from Spain and the United Kingdom, described the formation of a novel disulphide bond between C705 and C311–C320 accompanied by reduced cell surface expression and transport function (Gimenez and others 2012). GlyT2 transport activity depends on an E3 ubiquitin ligase LNX1/2 (ligand of numb protein X1/2). The N-terminal ring-finger domain of LNX1/2 enables GlyT2 ubiquitination at C-terminally localized lysines (de la Rocha-Munoz and others 2019). A recent study on GlyT2 SD mutants A277T and Y707C provided evidence for a strategy to overcome ER retention. The tight association of GlyT2 mutated proteins with the ER chaperone calnexin was suggested to hinder ER exit. This effect was rescued by overexpression of N-arachidonoyl glycine, possibly preventing the chaperone-transporter interaction and thus improving ER exit and membrane integration. Therefore, N-arachidonoyl glycine and its derivatives represent potential for novel pharmacotherapies for at least some mutations associated with GlyT2 ER retention (de la Rocha-Munoz and others 2021).

### Gain-of-Function Mutations

Interestingly, both gain- and loss-of-function mutations in GlyR subunits result in SD. Clinical symptoms in individuals carrying gain-of-function mutations do not differ from patients with loss-of-function mutations. For GlyRα1, gain-of-function mutations associated with SD have been found distributed along the entire GlyRα1 sequence—for example, I43F, Y128C, W170S, Q226E, V280M, P366L, and R414H (Fig. 3A–C, Table 2; Schaefer and others 2018a). As a main characteristic, spontaneous ion channel opening in the absence of glycine has been described. Moreover, prolonged decay times and inhibitory postsynaptic current durations have been noted for gain-of-function mutations (Langlhofer and others 2020; Zhang and others 2016). Spontaneously active channels have also been found for the de novo mutation GlyRβ^L285R^, which destabilizes the channel closed state (James and others 2013). The major question is whether prolonged open or decay times leading to an increased intracellular chloride concentration can explain SD symptoms. Intra- and extracellular Cl^-^ levels are typically regulated by the transporter KCC2, which is not fully functional after birth. Interestingly, reduced KCC2 function has been shown to result in a reduction in the number and size of GlyRα1 dendritic clusters without affecting GlyRα2 and GABA_A_R clusters (Schwale and others 2016). Since GlyRα2 is downregulated and replaced mainly by GlyRs containing α1β after birth in the spinal cord, GlyRα1 gain-of-function mutations leading to a temporally enhanced intracellular chloride ion concentration may reduce the overall number and size of the synaptically expressed α1-containing GlyRs, resulting in SD symptoms. Since both gain-of-function mutations have recently been reported for *GLRA2* (Chen and others 2022; Zhang and others 2017) and *GLRB* (Piro and others 2021), the underlying pathomechanisms for GlyR mutations are clearly more complex than previously considered.

## Novel Mutations in *Glra1, Glrb*, and *Slc5a6*

Novel GlyRα1 subunit mutations discovered in patients or mice with SD in the last 5 y include D70N, Q177K, L224X, R316X, P366L, A384P, and I401N (Fig. 3A–C, Table 2). Among them, D70N/R316X in a compound heterozygous form and I401N as a single mutation or in a compound heterozygous form with L224X have not been functionally characterized (Zhan and others 2020; Zhang and others 2020).

The murine GlyRα1 mutation Q177K and the human variant P366L have been extensively studied with single alterations that for themselves were unable to explain the SD phenotype. Both GlyRα1 mutants are examples of the complexity of SD since they cause small but significant impairments in glycinergic neurotransmission. These GlyRα1 variants are detailed in the next section (Langlhofer and others 2020; Schaefer and others 2017; Schaefer and others 2018a).

The first monozygotic twins with SD homozygous for an intronic *GLRB* splice site mutation (IVS5+5G→A) were recently identified (Estevez-Fraga and others 2020). This mutation had previously been described demonstrating that the intronic IVS5 mutation decreased splicing efficiency of *GLRB* exon 5 (Rees and others 2002). The homozygous carriers of IVS5+5G→A therefore represent the first human correlate of the spontaneous mouse mutant *spastic*. In *spastic*, an intronic LINE1 element insertion resulted in aberrant splicing and significantly reduced GlyRβ levels (Schaefer and others 2012). Homozygous *GLRB* (R190X) was identified in a 28-d-old infant with massive stiffness episodes and a nonhabituating nose-tap response. At the age of 4.5 y, intermittent bouts of tonic spasms still required enhanced doses of clonazepam for treatment (Table 3; Gupta and others 2020).

New GlyT2 mutations have been reported in a novel compound heterozygous case exhibiting a decline in severity of symptoms and frequency with age (S477Ffs9X/S477P; Dafsari and others 2019). A novel case with a P429L missense mutation included severe neuromotor deficits (Kitzenmaier and others 2019), as supported by functional studies demonstrating that GlyT2^P429L^ was expressed at the cell surface but nonfunctional in terms of glycine uptake.

## SD: An Increase in Complexity

To explain SD symptoms, unaffected expression and small functional changes are insufficient. New knowledge on the following has enhanced our current knowledge of SD pathomechanisms at the molecular level: mutants causing spontaneous opening, prolonged open times with subsequent temporal increases in intracellular chloride levels, changes in protein-protein interactions with identification of novel GlyR interactors, and the importance of presynaptic homomeric GlyRs.

### Presynaptic GlyRs

Research on GlyRα1 subunit mutations has mainly focused on defects in postsynaptic GlyRs. Presynaptic GlyRs were first identified in the medial nucleus of the trapezoid body in the brainstem of rodents (Turecek and Trussell 2001). By using two mouse models harboring GlyRα1^S271Q^ or α1^M287L^, GlyRα1 homomers were identified at presynaptic terminals in the Calyx of Held, a component of the brainstem auditory circuitry. Since Calyx of Held synapses lack other GlyR subunits, such as α2, α3, and β, the significantly lower glycine-mediated maximal currents, lower frequencies, and reduced glycine affinity argue for a contribution of defective presynaptic GlyRs to SD (Fig. 2; Xiong and others 2014). Intraperitonal injections of dehydroxycannabidiol (DH-CBD) significantly suppressed the acoustic and tactile-induced exaggerated startle response and hindlimb clasping in mutant mice. However, the effects of intraperitoneal injections of DH-CBD might also be mediated by receptor types and not specifically by GlyRs. Moreover, in vitro patch clamp recordings suggested that homomeric GlyRs exhibit a significantly enhanced sensitivity to DH-CBD as compared with heteromeric GlyRs, arguing that DH-CBD has capacity by targeting presynaptic GlyRs (Xiong and others 2014).

### Role of the β Subunit in Rescuing Glycinergic Function at Postsynaptic GlyRs

Concentrating on the different contribution of pre- and postsynaptic GlyRs to the SD phenotype, Zou and others (2019) investigated mutated GlyRs (α1^R271Q^, α1^S267Q^, α1^M287L^) in the presence of neighboring GABA_A_ (α1β2γ2) receptors. Interestingly, mutated GlyRs appeared to hijack GABA_A_ receptors at pre- and extrasynaptic sites, leading to reduced glycine/GABA release from presynaptic sites and thereby reducing glycinergic and GABAergic inhibition. Coexpression with the GlyRβ subunit resembling postsynaptic GlyRs prevented the interactions of mutated GlyRα1 with GABA_A_ receptors. Treatment with diazepam enhances the function of GABA_A_ receptors but also coassembled GABA_A_Rs/GlyRs (Fig. 2). By using DH-CBD on coassembled GABA_A_Rs/GlyRs, the function of mutated GlyRα1 was rescued. A disruption of the protein-protein interaction between GlyRs and GABA_A_Rs was suggested to underlie this effect (Zou and others 2020).

GlyRβ is, however, not always able to fully rescue the functional impairment of mutated GlyRα1 subunits at postsynaptic sites. Homomeric GlyRα1^S270T^ receptors exhibited more pronounced effects when the response was measured at glycine equilibrium than α1β heteromers. However, at the single-channel level, homomeric and heteromeric mutant GlyR configurations showed faster deactivation following fast synaptic-like applications of glycine, arguing that at the synaptic level both receptor configurations were affected in a similar manner (Wu and others 2020).

A partial rescue of some functional alterations has also been observed for the missense mutation α1^A384P^ when coexpressed with GlyRβ. GlyRα1^A384P^ results in a substantially higher desensitization level and lower agonist sensitivity when expressed homomerically. The incorporation of GlyRβ fully reversed reduced agonist sensitivity and partially reversed the enhanced desensitization of α1^A384P^. In comparison with wild type α1β, heteromeric α1^A384P^β showed enhanced desensitization but unchanged agonist-induced maximum responses, surface expression, ion channel conductance, and voltage dependence (Wang and others 2018). The compensatory effect of GlyRβ suggests that the SD phenotype in this individual might be due to a pronounced presynaptic defect rather than a postsynaptic deficit.

### SD Phenotypes: A Spectrum with Small but Additive Effects

One example for a highly complex molecular pattern with several smaller effects contributing to a SD phenotype came from the dominant human GlyRα1 mutation P366L localized in the ICD. While no alterations in trafficking, only slightly decreased maximal currents, and no change in the glycine dose-response curve were identified in vitro in transfected cells, the mutant α1^P366L^ demonstrated enhanced desensitization kinetics. In addition, electrophysiological recordings with artificial synapses between murine spinal cord neurons and transfected HEK293 cells revealed a reduced unitary conductance accompanied by spontaneous channel openings. P366L is localized in a poly-proline II helix representing a noncanonical Src homology 3 (SH3) recognition motif important for protein-protein interaction with syndapin I, an F-BAR domain protein involved in membrane remodeling. This poly-proline II helix also binds to the pleckstrin homology domain of collybistin and might contribute to postsynaptic anchoring (Breitinger and others 2021). The SH3 recognition motif is disrupted in α1^P366L^ and thus changes the endogenous syndapin I distribution, as demonstrated in cultured hippocampal neurons (Fig. 2; Langlhofer and others 2020).

By contrast, the mouse GlyRα1 mutant Q177K results in a lethal phenotype in homozygous *shaky* animals. Glycinergic dysfunction in *shaky* mice appears 1 wk in advance to the onset of SD symptoms but is in line with onset of GlyRα1 expression (Fig. 2; Schaefer and others 2018b). A slightly reduced surface expression and the rightward shift in glycine EC_50_ observed in transfected cells did not fully explain SD symptoms and lethality. Recordings from brainstem slices before and after onset of disease symptoms, however, demonstrated significantly decreased mIPSC amplitudes (miniature inhibitory postsynaptic current), frequency, and altered decay time constants. The in vivo expression level revealed a compensatory increased GlyRα1 expression. As a consequence, enhanced expression of presynaptic α1^Q177K^ in homozygous *shaky* mice favors a significant contribution of defective presynaptic GlyRs to SD in the affected animals (Schaefer and others 2017; Schaefer and others 2018b).

GlyRα1^Q177K^ is localized in the extracellular β8-β9 loop, which has been assigned an important determinant of structural transitions during channel gating (Du and others 2015). Indeed, α1^Q177K^ revealed a disrupted hydrogen bond network in the surrounding of residue 177 with the ligand-binding residue R65 (Janzen and others 2017; Schaefer and others 2017). Taken together, the detailed analysis of the *shaky* mouse model demonstrated not only how structural information can explain functional alterations but also the need for functional investigations in vivo or ex vivo in brain slices to fully understand glycinergic dysfunction in SD.

### Intracellular Protein-Protein Interaction

The human SD mutation α1^P366L^ interferes with syndapin I binding. First indications for binding of the GlyR poly-proline II helix to syndapin I came from studies on the corresponding region in GlyRβ (^435^KPPPAKP; Del Pino and others 2014). By using peptide arrays and tandem mass spectrometry–based analysis, a novel low-affinity interaction of GlyRα1 with syndapin I was verified, which was almost absent in α1^P366L^. Neurites expressing α1^P366L^ displayed a slight decrease in syndapin I intensity while the intensity significantly increased at the neuronal soma (Langlhofer and others 2020). Thus, disrupted GlyR–accessory protein interactions can also contribute to the molecular pattern underlying SD.

In vivo, the interaction of the GlyRβ subunit with the synaptic scaffolding protein gephyrin is essential for synaptic localization of GlyRs (Maynard and others 2021). To date, no GlyRβ subunit mutations have been found that localize to the gephyrin-binding motif in the GlyRβ ICD. A recent study investigated novel SD mutations in GlyRβ TMs (Y252S, S321F, and A455P; Fig. 3D–F, Table 3) and their interaction with gephyrin. In the presence of gephyrin, GlyRβ intracellular aggregate numbers (Y252S) were reduced or increased in area and perimeter (S321F and A455P). Both observed effects argue for a strong interaction with gephyrin underlying these intracellular accumulations. Transfection of hippocampal neurons confirmed differences in GlyR-gephyrin clustering for Y252S and A455P, leading to a significant reduction in GlyRβ-positive synapses. Structural changes within the GlyRβ subunit appear to underlie alterations in GlyRβ-gephyrin interactions, revealing new pathomechanisms for *GLRB* mutations (Fig. 2; Piro and others 2021).

Taken together, multiple evidence exists that SD results from the contribution of pre-, extra-, and postsynaptic effects. The coexpressed β subunit in vivo can rescue the postsynaptic deficits in GlyRα1 subunits, at least to some extent. Furthermore, if the GlyRβ subunit is the affected subunit, the interaction with intracellular binding partners might be altered and thus contribute to SD pathology.

### Other Possible Genes as Further Candidates for SD

Several individuals still lack a confirmed genetic diagnosis for SD (Davies and others 2010), and although mutations in gene promoters or regulatory elements of known genes have not been excluded, this has led to speculation that other SD genes may remain to be identified.

For example, *slc7a10* knockout mice lacking a functional Asc-1 (Figs. 1 and 2) revealed excessive startle responses, increased righting time, hindleg clasping, increased tremors, and reduced activity in open field tests consistent with other mouse models of SD (Safory and others 2015). Asc-1 is an Na^+^-independent plasma membrane transporter present in neurons and astrocytes with high affinity for neutral small amino acids, such as glycine, L-serine, D-serine, alanine, and cysteine. Asc-1 is highly expressed in the brain, caudal brainstem regions, and spinal cord close to high densities of glycinergic activity (Helboe and others 2003). The role of Asc-1 in maintaining neuronal presynaptic glycine levels suggests that *SLC7A10* is a plausible candidate gene for genetically undiagnosed cases of human SD (Ehmsen and others 2016). However, to date, no *SLC7A10* mutations in human patients with SD have been described. The role of Asc-1 in determining presynaptic glycine levels suggests that, like GlyT2 mutations, Asc-1 mutations may alter respiratory pattern formation (Mesuret and others 2018) or cause neonatal apnea episodes.

*SLC6A9* encoding GlyT1 is predominantly expressed in glial cells and facilitates rapid clearance of glycine from the synaptic cleft. *Slc6a9* knockout mice die within a few hours after birth, most likely due to respiratory deficiencies (Gomeza and others 2003). Interestingly, GlyT1 mutations have recently been reported in a human disorder termed GlyT1 encephalopathy, characterized by severe hypotonia and startle-like responses provoked by sudden loud noises and tactile stimulation. Individuals with GlyT1 mutations also exhibit respiratory failure requiring mechanical ventilation, encephalopathy, impaired consciousness, and unresponsiveness (Hauf and others 2020; Mademont-Soler and others 2021).

Mutations in *CTNNB1* encoding β-catenin, a protein known to play a role in cell adhesion, have been depicted in patients with a range of neurodevelopmental disorders (intellectual disability, microcephaly, and other syndromic features) but who also exhibited hyperekplexia and episodic falls when startled by noise or touch (Zhan and others 2021).

Two recent reviews pointed out additional genes as candidates for SD (Saini and Pandey 2020; Zhan and others 2021). Among them are *SLC32A1* encoding VIAAT, *SLC6A17* encoding Rxt1/NTT4, and *ATAD1* encoding a thorase. There are no studies linking genetic defects in these three genes with human patients with SD.

*SLC32A1* was previously considered a candidate gene for human hyperekplexia, but eight missense variants in *SLC32A1* have recently been reported in families with genetic epilepsy with febrile seizures plus or idiopathic generalized epilepsy (Heron and others 2021), making it unlikely that mutations in *SLC32A1* are associated with SD.

NTT4 transports proline, glycine, leucine, and alanine and has been mainly found at glutamatergic synapses and some GABergic neurons. In addition, individuals with missense mutations in *SLC6A17* presented with moderate to severe intellectual disability, a progressive tremor, speech impairment, and behavioral problems (Iqbal and others 2015). Taken together, this evidence eliminates *SLC6A17* from future consideration as a candidate gene for human SD.

*ATAD1* mutations have been linked to SD (hyperekplexia 4). Hyperekplexia 4 shares phenotypic similarities to individuals with hyperekplexia, but inhibitory neurotransmission is not affected. Instead, the affected thorase alters AMPA receptor recycling by forcing the disassembly of the AMPA receptor-binding protein, GRIP1, and the AMPA receptor (Ahrens-Nicklas and others 2017). We consider this to be a genetically distinct disorder, and use of the term *hyperekplexia* is misleading, since inhibitory neurotransmission is not affected and clonazepam would not be expected to be a useful pharmacotherapy.

## Altered Glycinergic Neurotransmission in Additional Disease Phenotypes

### Fear and Anxiety

Individuals with SD have been described as more anxious. This was presumed to be due to the possibility of uncontrolled falls following unexpected auditory or tactile stimuli. A further link between enhanced anxiety and altered glycinergic inhibition was provided by a genome-wide association study that discovered single-nucleotide polymorphisms (SNPs) in *GLRB* in individuals with a prevalence for agoraphobia (Fig. 2; Deckert and others 2017). These SNPs are noncoding and assumed to modify GlyR expression. Behavioral studies in heterozygous *spastic* mice with reduced GlyRβ levels but no SD phenotype demonstrated a significantly decreased time that affected mice spent in the open field as compared with time spent along the walls, similar to *GLRB* SNP carriers with enhanced agoraphobia (Deckert and others 2017). However, other behavioral tests (e.g., elevated plus maze, dark/light) lacked significant differences between mice with reduced GlyRβ levels and wild type controls. Therefore, a modulatory function for the GlyRβ subunit within glycinergic circuits in neuronal networks is important for fear and fear-related behaviors (Schaefer and others 2020).

### Stiff Person Syndrome

Patients experiencing stiff person syndrome or progressive encephalitis with rigidity and myoclonus exhibit excessive startle responses and muscle spasms similar to patients with SD. In contrast to the genetic origin of SD, patients with stiff person syndrome harbor autoantibodies against GlyR subunits. Our molecular understanding of the molecular pathomechanisms of GlyR autoantibodies include the following: 1) enhanced GlyR internalization upon crosslinking of membrane-expressed GlyRs followed by lysosomal degradation, 2) activation of complement, and 3) blockade of GlyR function upon antibody binding. Moreover, the pathological potential of the autoantibodies was demonstrated following autoantibody transfer into zebrafish larvae, impairing the escape response of the animal similar to SD mutations introduced in zebrafish, which is compatible with an abnormal startle response in patients with stiff person syndrome or progressive encephalitis with rigidity and myoclonus (Fig. 2; Carvajal-Gonzalez and others 2014; Rauschenberger and others 2020).

## Insights from Molecular Structures of GlyRs

Until 2015, no X-ray or cryo-EM structures of the GlyR were available. First insights into the organization of cys-loop receptor ECDs came from the X-ray structure of the homologous acetylcholine-binding protein isolated from *Lymnaea stagnalis* (Brejc and others 2001). The cryo-EM structures of the zebrafish GlyRα1 and the X-ray structure of human GlyRα3 revolutionized our current knowledge on GlyR receptor states, such as the open/closed and desensitized/partially desensitized configurations. In addition, residues involved in structural transitions following ligand binding to ion channel opening as well as residues participating in neurosteroid, picrotoxin, and anesthetic binding have been identified (Fig. 2; Du and others 2015; Huang and others 2015). These structures provided significant impact on additional interpretations of observed physiological changes for SD mutants and their contributions to the disease phenotype. The strychnine-bound GlyR structure revealed electrostatic attraction of Q226E in TM1 to R271 in TM2 of the adjacent subunit, causing a tilt of TM2 away from the pore axis and explaining the constitutive open channel observed for this SD mutation. The ivermectin/glycine-bound GlyR illuminated the role of SD mutations localized at the ECD–TM domain interface and how SD mutations such as R218Q in pre-M1 or Y279C in the TM2-3 loop disturb their interactions with residues in the cys-loop followed by transitional block upon agonist binding. The glycine-bound open GlyR structure clarified the modified interactions between adjacent pore-lining mutated residues in SD (e.g., Q266H and R271Q/L) and their influence on ion channel properties. Moreover, these structures enabled predictions of altered stacking interactions within TM and between TM regions for novel GlyRβ SD mutations and their functional consequences (Piro and others 2021). The correct GlyR stoichiometry has been a long debate. The recently solved cryo-EM structures of heteromeric GlyRs suggest a 4α:1β stoichiometry (Yu and others 2021; Zhu and Gouaux 2021) and will shed light on our interpretations and understanding of postsynaptic changes at glycinergic synapses under healthy and SD conditions.

However, almost all structures lack the large ICD where some SD mutations are localized. The ICD was replaced by a short peptide sequence present in bacterial cys-loop receptor homologs GLIC and ELIC. Kumar and others (2020) used the full-length human GlyRα1 for structural investigation, but the ICD was unstructured except for small domains following TM3 and before TM4, which showed α-helical arrangements. Similar observations have been made following the analysis of the cryo-EM structure of heteromeric GlyRs (Zhu and Gouaux 2021). The largely disordered ICD might also represent a consequence of the lack of intracellular postsynaptic proteins during structural investigations. Even if unfolded or disordered, the ICD structure is essential to understand intracellular receptor modifications induced by SD mutations at the ECD.

The structure of GlyT2 has still not been resolved. The X-ray structure of the GlyT1 transporter was very recently solved with a resolution of 3.4 Å (Shahsavar and others 2021). GlyT1 has a 50% homology to GlyT2 at the nucleotide and amino acid level. As such, the solved GlyT1 structure will now allow more precise interpretations of GlyT2 SD mutation pathomechanisms.

Another obvious gap is the missing X-ray structure of full-length gephyrin. Researchers have focused on the structure of the gephyrin E-domain, which is responsible for the interaction with defined motifs in the ICD of GlyRβ or GABA_A_ receptor subunits α1, α2, and α3 (Maric and others 2017). Protein-protein interactions involve short linear motifs. The use of alternative approaches, such as temperature-related intensity change, allowed the detection of low- and high-affinity protein-peptide interactions between gephyrin and GlyRβ validated by binding to recombinant and native GlyRs (Maric and others 2017; Schulte and others 2021). Although such methods have limitations, the offered high throughput of temperature-related intensity change allows a rapid detection of protein-binding profiles. Such studies that elucidated the protein interactions with different affinities between gephyrin and the GlyR enhanced our knowledge on synaptic exchange rates, receptor fields, and dynamics and are essential to further understand cooperative influences of extracellular GlyR mutations to the binding of intracellular protein partners, which may differ under disease conditions such as SD.

## Future Directions to Understand Disease Complexity

Recent findings on GlyRα1 and β SD mutations, although located in the ECD or TM domains, have pointed toward alterations of GlyR–accessory protein interactions, which in turn affect glycinergic function. How extracellular or TM mutations influence intracellular protein-protein interactions remains elusive, but in our view, this is likely to be linked to changes in GlyR conformation. Moreover, our current knowledge on receptor-lipid interactions and their relevance for stabilization of receptor states and receptor gating transitions is still limited. Finally, the use of three-dimensional (3D) cell culture models might reflect a suitable alternative to study disease mechanisms of rare diseases and their molecular pathologies.

The GlyR ICD lacks cryo-EM densities (Figs. 2 and 3; Zhu and Gouaux 2021). Similar findings have been observed for the closely related heteromeric GABA_A_R (α1β3γ2L) demonstrating a disordered TM3-4 loop, which likely reflects a lack of interacting postsynaptic proteins (Laverty and others 2019). Therefore, the solved GlyR structures obtained to date are unable to provide hints for structural transitions transmitted from an ECD mutation toward intracellular binding partners. Thus, there is still an important gap between structural knowledge and findings from in vitro molecular, cellular, and protein biochemical approaches as well as physiological assessments.

Protein-protein interaction as the tight association between GlyRβ (residues 378–426) and gephyrin and its necessity for synaptic localization is well characterized (Schrader and others 2004). The binding between gephyrin and GlyRβ is bimodal with a high- and a low-affinity binding sites. The residues responsible for the high-affinity gephyrin-GlyRβ interaction have been mapped to C-terminal residues of the GlyRβ binding core (residues 394–413), while the low-affinity binding site is formed by N-terminal residues within the binding core. High-affinity gephyrin binding is involved in GlyR confinement at synaptic and extrasynaptic sites. Low-affinity binding rather contributes to GlyR confinement by fine-tuning synaptic clusters (Grunewald and others 2018). High- and low-affinity binding of gephyrin to GlyRs is in line with the dynamic organization of different subpopulations at inhibitory synapses, a tight interacting receptor-scaffold domain and a more loosely bound receptor scaffold population, of reciprocally stabilized GlyR and gephyrin proteins (Chapdelaine and others 2021). By using high-resolution microscopy techniques, such as single-molecule localization microscopy and correlative light and electron microscopic analysis that enable high spatial resolution, the GlyR-scaffold occupancy and receptor densities at glycinergic synapses have been investigated. A constant packing of 2000 GlyRs µm^-2^ at spinal cord synapses throughout adulthood has been estimated. This number of GlyRs per synapse did not change in analysis of heterozygous *oscillator* mice that lacked 50% of the GlyRα1 subunit. A significant decrease was found in synapse size in heterozygous *oscillator* animals, arguing that under disease conditions the morphology and size of glycinergic synapses might play a key role in governing glycinergic postsynaptic strength in spinal cord circuits (Maynard and others 2021).

Furthermore, the protein-protein interaction between GlyRβ and syndapin I as well as between GlyRα1 and syndapin I but with lower affinity seems to be important for glycinergic synapse organization (Del Pino and others 2014; Langlhofer and others 2020). Similar to GlyRβ decoupled from gephyrin, syndapin 1 knockout increases GlyRβ mobility. Yet, gephyrin decoupling from GlyRs increases GlyR-syndapin interactions and thus regulates fine-tuning of synaptic efficacy at inhibitory synapses (Troeger and others 2021). Thus, gephyrin and syndapin I seem to cooperate in receptor scaffolding functions, regulating the size and density of GlyR clusters. It also seems clear from recent GlyR SD mutations that the impairment of this postsynaptic fine-tuning underlies or at least contributes to SD clinical phenotypes.

Besides protein-protein interaction, protein-lipid interaction might interfere and/or stabilize receptor states. Lipid densities have been detected close to TM domains on the extra- and intracellular sides of the bilayer (Fig. 2; Laverty and others 2019; Zhu and Gouaux 2021). Molecular dynamic simulations for the GlyR have shown that phosphatidylcholine interacts with receptor regions involved in structural transitions during the gating process, such as loop C, pre-TM1, β8-β9 loop, β1-β2 loop, cys-loop, and TM2-3 linker. In contrast, phosphatidylserine has been found closer to the intracellular side bound to TM3 and TM4 and coincides with the PIP2 binding site (phosphatidylinositol-4,5-biphosphate; Damgen and Biggin 2021). As lipids might interfere with receptor gating, the identification of protein-lipid interaction sites may become more important in the future as a prerequisite to design lipid-like allosteric drugs.

Three-dimensional cell culture models to study disease mechanism such as SD may become an important tool in the future. Three-dimensional cell culture models can be set up from various neuronal subtypes and can be easily targeted by viral infections to introduce SD mutations of interest. So far, SD mutations are rarely studied in a neuronal context and then only in 2-dimensional (2D) monolayers. Between in vitro 2D cell cultures and in vivo experiments, there is a large gap that might get filled by investigations of 3D cell culture disease models. The importance of the third dimension has been pointed out in a 3D spinal cord neuronal model comparing synapse development in 2D with 3D (Fig. 2; Fischhaber and others 2021). This model suggests that synaptic maturation and network formation in 3D cultures are faster. A critical mass of interacting neurons is, however, essential to detect disease phenotypes and study protein-protein interactions in the 3D context. However, there are still several issues to overcome until such 3D disease models will represent suitable tools and an alternative for animal experiments. In 3D, reproducible hydrogel compositions resembling the native extracellular matrix need to be defined that allow neuronal differentiation and possibly redifferentiation of neurons from patient-specific stem cells.

## Conclusion

This review provides an update on novel SD mutations within the last 5 y. Increasing knowledge at the structural level for homomeric and heteromeric GlyRs and new mouse models have enhanced top-down structure-function approaches for SD mutations. For future investigations, main directions can be defined as follows: the identification of mechanisms that underlie alterations of intracellular interactions with GlyR accessory proteins by extra- or intracellular SD mutations, the study of GlyR-protein/lipid interactions and how they are affected by SD mutations, the precise contribution of presynaptic GlyRs to SD phenotypes, the exploration of pharmacotherapies suitable for overcoming SD mutations resulting in trafficking defects for GlyRs or GlyTs, and the establishment of suitable 3D cell culture models to further ascertain our molecular understanding of SD mutations.
